# Cell-autonomous mechanisms of chronological aging in the yeast *Saccharomyces cerevisiae*

**DOI:** 10.15698/mic2014.06.152

**Published:** 2014-05-27

**Authors:** Anthony Arlia-Ciommo, Anna Leonov, Amanda Piano, Veronika Svistkova, Vladimir I. Titorenko

**Affiliations:** 1 Department of Biology, Concordia University, Montreal, Quebec H4B 1R6, Canada.

**Keywords:** yeast chronological aging, systems biology of cellular aging, cell-autonomous mechanisms of longevity regulation, proteostasis, lipid metabolism, mitochondria, carbohydrate metabolism

## Abstract

A body of evidence supports the view that the signaling pathways governing cellular aging - as well as mechanisms of their modulation by longevity-extending genetic, dietary and pharmacological interventions - are conserved across species. The scope of this review is to critically analyze recent advances in our understanding of cell-autonomous mechanisms of chronological aging in the budding yeast *Saccharomyces cerevisiae*. Based on our analysis, we propose a concept of a biomolecular network underlying the chronology of cellular aging in yeast. The concept posits that such network progresses through a series of lifespan checkpoints. At each of these checkpoints, the intracellular concentrations of some key intermediates and products of certain metabolic pathways - as well as the rates of coordinated flow of such metabolites within an intricate network of intercompartmental communications - are monitored by some checkpoint-specific ʺmaster regulatorʺ proteins. The concept envisions that a synergistic action of these master regulator proteins at certain early-life and late-life checkpoints modulates the rates and efficiencies of progression of such processes as cell metabolism, growth, proliferation, stress resistance, macromolecular homeostasis, survival and death. The concept predicts that, by modulating these vital cellular processes throughout lifespan (i.e., prior to an arrest of cell growth and division, and following such arrest), the checkpoint-specific master regulator proteins orchestrate the development and maintenance of a pro- or anti-aging cellular pattern and, thus, define longevity of chronologically aging yeast.

## INTRODUCTION

The budding yeast *S. cerevisiae* is an advantageous model organism for unveiling fundamental mechanisms and biological principles underlying the inherent complexity of cellular aging in multicellular eukaryotes 1,2,3,4,5,6. Because this unicellular eukaryote is amenable to comprehensive biochemical, genetic, cell biological, chemical biological, system biological and microfluidic dissection analyses 7,8,9,10,11,12, its use as a model in aging research provided deep mechanistic insights into cellular processes essential for longevity regulation in evolutionarily distant eukaryotic organisms. Due to the relatively short and easily monitored chronological and replicative lifespans of the yeast *S. cerevisiae*, it played a pivotal role in discovering: (1) numerous genes that impact cellular aging and define organismal longevity not only in yeast but also in eukaryotic organisms across phyla; (2) some key nutrient- and energy-sensing signaling pathways that orchestrate an evolutionarily conserved set of longevity-defining cellular processes across species; and (3) several aging-decelerating and longevity-extending small molecules, many of which slow down aging, improve health, attenuate age-related pathologies and delay the onset of age-related diseases in evolutionarily distant multicellular eukaryotic organisms 1,2,3,4,5,6,13,14,15,16,17,18,19,20,21,22. These studies convincingly demonstrated that the signaling pathways governing cellular aging and mechanisms of their modulation by longevity-extending genetic, dietary and pharmacological interventions are conserved across species.

There are two different paradigms of yeast aging. Each of them is traditionally investigated separately from each other with the help of robust assays. These assays are conducted under controllable laboratory conditions 23,24,25,26 and have been recently automated to enable a systems-level analysis of the aging process in a high-throughput format 9,10,11,12,27,28,29. In the chronological aging paradigm, yeast aging is defined by the length of time during which a cell remains viable after an arrest of its growth and division 23,30,31. Yeast chronological aging under laboratory conditions is assessed using a simple clonogenic assay. This assay measures the percentage of yeast cells that in liquid cultures remain viable at different time points following entry of a cell population into the non-proliferative stationary phase. Cell viability in the clonogenic assay is assessed by monitoring the ability of a cell to form a colony on the surface of a solid nutrient-rich medium 3,25,30. Chronological aging in yeast is believed: (1) to mimic aging of non-dividing, post-mitotic cells (such as neurons) in a multicellular eukaryotic organism; and (2) to serve as a simple model for organismal aging 32,33. In the replicative aging paradigm, yeast aging is defined by the maximum number of daughter cells that a mother cell can produce before becoming senescent 24,34,35. *S. cerevisiae* reproduces by asymmetric cell division; therefore, its replicative aging under laboratory conditions is typically assessed by using a micromanipulator to remove the budding progeny of a mother cell and counting the cumulative number of asymmetric mitotic divisions this mother cell could undergo 24,35. Replicative aging in yeast is thought to mimic aging of dividing, mitotically active cells (such as lymphocytes) in a multicellular eukaryotic organism 2,32. The use of robust assays for elucidating longevity regulation in chronologically or replicatively aging yeast under controllable laboratory conditions has significantly advanced our understanding of cell-autonomous mechanisms that orchestrate longevity-defining cellular processes within an individual cell in eukaryotic organisms across phyla 1,2,3,5,6,10.

Recent studies in yeast also advanced fundamental knowledge about cell-non-autonomous intraspecies mechanisms of longevity regulation. Such mechanisms operate within organized populations of yeast cells that are attached to solid surfaces to form a colony or a biofilm; these cells: (1) communicate with each other and cells in surrounding colonies or biofilms; (2) age chronologically and replicatively; and (3) undergo spatially organized growth, differentiation, aging or death, depending on their position within the colony 4,36,37,38,39,40,41,42,43.

It seems that cell-autonomous and cell-non-autonomous intraspecies mechanisms regulating yeast longevity have evolved in the process of natural selection within an ecosystem 44,45,46. It has been recently proposed that this process: (1) is governed by ecosystemic interspecies mechanisms of lifespan regulation operating within the ecosystem; and (2) is driven by the ability of yeast cells to undergo specific pro-survival changes in their metabolism and physiology in response to some chemical compounds that, after being released to the ecosystem by other groups of organisms, may trigger a hormetic and/or cytostatic response in yeast 44,45,46,47,48.

In this review we analyze recent progress in understanding of cell-autonomous mechanisms of chronological aging in the yeast *S. cerevisiae*. Our analysis suggests a concept of a biomolecular network underlying the chronology of cellular aging in yeast. This concept envisions that: (1) the network integrates the vital processes of cell metabolism, growth, proliferation, stress resistance, macromolecular homeostasis, survival and death; (2) the network progresses through a series of the early-life and late-life "checkpoints"; (3) a gradual progression of the network through these lifespan checkpoints is monitored by some checkpoint-specific "master regulator" proteins; and (4) such progression is critically important for establishing the pace of cellular aging.

## CELL-AUTONOMOUS MECHANISMS ORCHESTRATE LONGEVITY-DEFINING CELLULAR PROCESSES IN CHRONOLOGICALLY AGING YEAST PRIOR TO AN ARREST OF CELL GROWTH AND DIVISION

A body of recent evidence supports the view that certain cellular processes taking place early in life of a chronologically aging yeast cell, prior to entry into a non-proliferative state, define the length of time during which this cell remains viable after such entry - i.e., define longevity of chronologically aging yeast grown under controllable laboratory conditions in liquid media 3,20,23,49,50,51,52,53,54,55,56,57,58,59,60,61,62,63,64,65,66,67,68,69,70,71,72,73. These longevity-defining cellular processes: (1) are essential for metabolism, growth, proliferation, stress resistance, macromolecular homeostasis, survival and death of individual yeast cells that age chronologically; and (2) are orchestrated via cell-autonomous mechanisms of lifespan regulation operating within these cells. The longevity-defining cellular processes and mechanisms orchestrating their progression in chronologically aging yeast prior to entry into a non-proliferative state (and, for some of them, after such entry) are outlined below in this section.

### Intracellular trehalose modulates cellular protein homeostasis (proteostasis)

**Figure 1:**
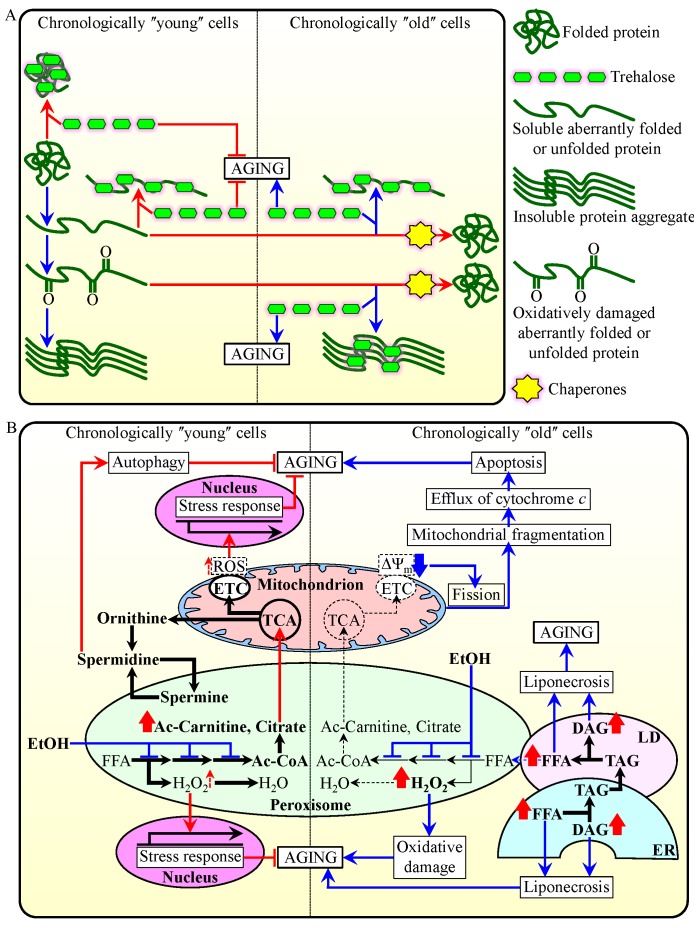
FIGURE 1: Cell-autonomous mechanisms define longevity of chronologically aging yeast by orchestrating trehalose metabolism and metabolic processes confined to peroxisomes. **(A)** A model for molecular mechanisms through which trehalose regulates the process of cellular aging in yeast by modulating protein folding, misfolding, unfolding, refolding, oxidative damage, solubility and aggregation in chronologically "young" and "old" cells. **(B)** A model for how the age-dependent efficiency of peroxisomal protein import in chronologically aging yeast defines the age-related metabolic pattern of peroxisomes, thus impacting longevity-defining processes in other cellular compartments and ultimately establishing a pro- or anti-aging cellular pattern. Activation arrows and inhibition bars denote pro-aging processes (displayed in blue color) or anti-aging processes (displayed in red color). Please see text for additional details. Ac-Carnitine, acetyl-carnitine; Ac-CoA, acetyl-CoA; DAG, diacylglycerol; ER, endoplasmic reticulum; ETC, electron transport chain; EtOH, ethanol; FFA, non-esterified ("free") fatty acids; LD, lipid droplet; ROS, reactive oxygen species; TAG, triacylglycerols; TCA, tricarboxylic acid cycle; ΔΨ_m_, electrochemical potential across the inner mitochondrial membrane.

Recent studies revealed that the intracellular concentrations of trehalose prior to cell entry into a non-proliferative state and following such entry play essential and differing roles in defining longevity of chronologically aging yeast. This is because trehalose is involved in modulating protein folding, misfolding, unfolding, refolding, oxidative damage, solubility and aggregation throughout lifespan 23,61 (Figure 1A). In chronologically "young" yeast cells, which undergo growth and division, trehalose plays an essential longevity-extending role because this non-reducing disaccharide: (1) binds to newly synthesized cellular proteins, thereby stabilizing their native folding states and attenuating their conversion into aberrantly folded and/or unfolded protein species; (2) shields the contiguous exposed hydrophobic side chains of amino acids that are abundant in misfolded, partially folded and unfolded protein species and that are known to promote their aggregation, thereby eliciting a direct inhibitory effect on the formation of insoluble protein aggregates; and (3) protects cellular proteins from oxidative carbonylation by interacting with their carbonylation-prone aberrantly folded species, thus having an indirect inhibitory effect on the aggregation of oxidatively damaged proteins 23,61 (Figure 1A).

In contrast, in chronologically "old" yeast cells, which do not grow or divide, trehalose plays a key role in shortening longevity. This is because in such cells trehalose shields the patches of hydrophobic amino acid residues that are abundant in aberrantly folded protein species 61. Thus, in chronologically "old" yeast cells trehalose competes with molecular chaperones for binding with these patches of hydrophobic amino acid residues known to be required for the chaperone-assisted refolding of misfolded, partially folded and unfolded protein species 74,75,76,77 - either soluble or extracted from protein aggregates with the help of molecular chaperones 61 (Figure 1A). Importantly, it has been demonstrated that a caloric restriction (CR) diet and certain genetic interventions affecting trehalose synthesis or degradation extend longevity of chronologically aging yeast because they simultaneously: (1) increase the intracellular concentration of trehalose by 70-160% (above its level detected in yeast cultured under non-CR conditions) in chronologically "young", proliferating cells; and (2) reduce the intracellular concentration of trehalose by 60-80% (below a threshold observed in yeast cultured under non-CR conditions) in chronologically "old", non-proliferating cells 61. These findings suggest the existence of at least two "checkpoints" during the lifespan of a chronologically aging yeast cell at which the intracellular concentration of trehalose (which depends on a balance between trehalose synthesis and degradation) defines its longevity by modulating cellular proteostasis. It seems that one of these checkpoints exists early in life of a chronologically aging yeast cell (i.e., prior to entry into a non-proliferative state), whereas the other checkpoint occurs late in its life (i.e., after such cell enters a non-proliferative state).

### Protein import into the peroxisome impacts longevity-defining processes in other cellular compartments

The efficiency of peroxisomal protein import has been shown to decline with the chronological age of a eukaryotic cell 57,58,78,79. Such import is driven by Pex5p and Pex7p, the peroxisomal targeting signal type 1 (PTS1) and PTS2 cytosolic shuttling receptors, respectively 80,81,82. Recent findings support the view that the age-dependent efficiency of protein import into the peroxisome defines the efficiencies of fatty acid oxidation, hydrogen peroxide turnover and anaplerotic metabolism within this organelle. These metabolic processes are known to modulate the dynamic communications of peroxisomes with other cellular compartments via a unidirectional or bidirectional flow of certain soluble metabolites and lipids 20,57,58,65,85,86,87,88,89. By influencing longevity-defining cellular processes confined to these other compartments, the metabolic processes within the peroxisome cause the development of a pro- or anti-aging cellular pattern 20,54,57,58,65,83,84,85,86,87,88,89.

Altogether, these findings suggest a model for how the age-dependent efficiency of peroxisomal protein import in chronologically aging yeast defines the age-related metabolic pattern of peroxisomes, thus impacting longevity-defining processes in other cellular compartments and ultimately establishing a pro- or anti-aging cellular pattern (Figure 1B). The model envisions that chronologically "young" yeast cells develop and maintain an anti-aging cellular pattern in part because in these cells the efficiency of Pex5p- and Pex7p-dependent peroxisomal protein import exceeds a threshold. Specifically, such "young" cells are proficient in peroxisomal import of the following proteins: (1) catalase Cta1p and peroxiredoxin Pmp20p, both required for decomposition of hydrogen peroxide and other reactive oxygen species (ROS) within the peroxisome; (2) Fox1p, Fox2p and Fox3p, enzymes involved in peroxisomal β-oxidation of fatty acids to acetyl-CoA; and (3) the citrate synthase Cit2p and acetyl-carnitine synthase Cat2p, both facilitating the replenishment of tricarboxylic acid (TCA) cycle intermediates destined for mitochondria by catalyzing the anaplerotic conversion of acetyl-CoA to citrate and acetyl-carnitine, respectively 14,20,57,58,85,90,91,92 (Figure 1B). The efficient peroxisomal import of all these proteins in chronologically "young" yeast cells enables the establishment of an anti-aging cellular pattern by: (1) minimizing the oxidative damage to peroxisomal proteins and membrane lipids; (2) maintaining the intracellular concentration of peroxisomally produced hydrogen peroxide at a threshold which is insufficient to damage cellular macromolecules but can activate transcription of nuclear genes essential for cell survival, thus promoting the longevity-extending cellular process of "stress-response hormesis"; (3) stimulating the TCA cycle and electron transport chain (ETC) in mitochondria, thus enabling to sustain mitochondrially generated ROS at a non-toxic level which is sufficient to stimulate transcription of nuclear genes encoding stress-protecting and other anti-aging proteins 20,23,57,58,65,92 (Figure 1B).

Noteworthy, peroxisomes in yeast house the polyamine oxidase Fms1p, an enzyme involved in the synthesis of spermidine 17,93. This natural polyamine has been shown to extend longevity of chronologically aging yeast by stimulating the essential cytoprotective cellular process of autophagy 13,94,95 (Figure 1B). Because the intracellular concentration of spermidine in chronologically "young" yeast exceeds that in chronologically "old" yeast 13, one could speculate that peroxisomal import of Fms1p early in life of a chronologically aging yeast cell is more efficient than it is late in life, after entry of a chronologically aging yeast cell population into a non-proliferative state. In this scenario, chronologically "young" yeast cells develop and maintain an anti-aging cellular pattern in part because they are proficient in peroxisomal import of a protein needed for the synthesis of a natural polyamine which promotes the longevity-extending cellular process of autophagy (Figure 1B).

Our model further posits that in chronologically "old" yeast cells the efficiencies of Pex5p- and Pex7p-dependent peroxisomal import of Cta1p, Pmp20p, Fox1p, Fox2p, Fox3p, Cit2p and Cat2p markedly decline (Figure 1B). Such deterioration of peroxisomal protein import below a threshold in these cells causes the development of a pro-aging cellular pattern by: (1) elevating the intracellular concentration of peroxisomally produced hydrogen peroxide above a cytotoxic level, thus increasing the extent of oxidative damage to cellular macromolecules; (2) reducing the efficiency of Fox1p-, Fox2p- and Fox3p-driven peroxisomal oxidation of fatty acids derived from triacylglycerols that are synthesized in the endoplasmic reticulum (ER) and deposited within lipid droplets (LD) - thus elevating the concentrations of non-esterified ("free") fatty acids and diacylglycerol, both of which are known to elicit an age-related form of liponecrotic programmed cell death (PCD); (3) diminishing the replenishment of TCA cycle intermediates destined for the TCA cycle in mitochondria - thus triggering a cascade of events that reduce the ETC in mitochondria, lower electrochemical potential across the inner mitochondrial membrane (IMM), promote mitochondrial fragmentation, cause the efflux of cytochrome *c* and other pro-apoptotic proteins from fragmented mitochondria, and ultimately elicit an age-related form of apoptotic PCD 14,20,23,54,57,58,65,84,91,92,96,97,98,99,100,101,102,103 (Figure 1B).

Furthermore, our model envisions that chronologically "old" yeast cells develop and maintain a pro-aging cellular pattern in part because they exhibit a low efficiency of peroxisomal import of the polyamine oxidase Fms1p, which is required for the synthesis of spermidine 13,17,93,94,95 (Figure 1B).

Noteworthy, both chronologically "young" and "old" yeast cells grown in a nutrient-rich medium under longevity-shortening non-CR conditions have been shown to accumulate ethanol, a product of glucose fermentation 23,84. Ethanol is known to repress the synthesis of Fox1p, Fox2p and Fox3p, thereby suppressing Fox1p-, Fox2p- and Fox3p-driven peroxisomal oxidation of fatty acids 90,104 (Figure 1B). By suppressing peroxisomal oxidation of fatty acids to acetyl-CoA in chronologically "young" cells under non-CR conditions, the accumulated ethanol attenuates the anaplerotic conversion of acetyl-CoA to citrate and acetyl-carnitine - thus inhibiting an aging-decelerating cellular process of the replenishment of TCA cycle intermediates destined for mitochondria 57,58,90,104 (Figure 1B). Moreover, by suppressing peroxisomal oxidation of fatty acids to acetyl-CoA in chronologically "old" cells under non-CR conditions, the accumulated ethanol also elevates the concentrations of non-esterified ("free") fatty acids and diacylglycerol - thus triggering an age-related form of liponecrotic PCD (Figure 1B) 14,23,54,57,58,84,103. It should be stressed that neither chronologically "young" nor chronologically "old" yeast cells grown under longevity-extending CR conditions amass ethanol 23,84. Such inability of yeast cells limited in calorie supply to accumulate ethanol is one of the reasons of why they are able to develop and maintain an anti-aging cellular pattern throughout lifespan 23,54,57,58,84 (Figure 1B).

In sum, it is conceivable that there are at least two checkpoints during the lifespan of a chronologically aging yeast cell at which the age-dependent efficiency of peroxisomal protein import defines the age-related metabolic pattern of peroxisomes, thus impacting longevity-defining processes in other cellular compartments and ultimately establishing a pro- or anti-aging cellular pattern. It seems that one of these checkpoints occurs early in life of a chronologically aging yeast cell (i.e., prior to an arrest of cell growth and division), whereas the other checkpoint exists late in its life (i.e., following such arrest).

### Coupled mitochondrial respiration, mitochondrial membrane potential and mitochondrial ROS production affect longevity-defining processes in other cellular locations

The functional state of mitochondria and mitochondrial ROS production early in life of a chronologically aging yeast cell, prior to entry into a non-proliferative state, have been shown to define the length of time during which this cell remains viable after such entry - i.e., define longevity of chronologically aging yeast 3,14,23,31,49,50,51,53,55,56,62,64,68,69,70,71,72,73,105,106,107,108. One key feature of the longevity-defining functional state of mitochondria in chronologically "young", proliferating yeast cells is the capacity of electron transport along the respiratory chain coupled to ATP synthesis 3,14,23,49,50,51,62,64,70,72,73,106,107. Another such key feature is the value of mitochondrial membrane potential; it depends on a balance between the capacities of ETC-driven proton transport from the matrix to the intermembrane space and proton translocation across the IMM in the opposite direction 3,14,23,50,51,56,64,70. Moreover, the longevity-defining process of mitochondrial ROS production in chronologically "young" yeast cells depends on the efficiency of coupling between the ETC and oxidative phosphorylation (OXPHOS) system in mitochondria 50,51.

**Figure 2:**
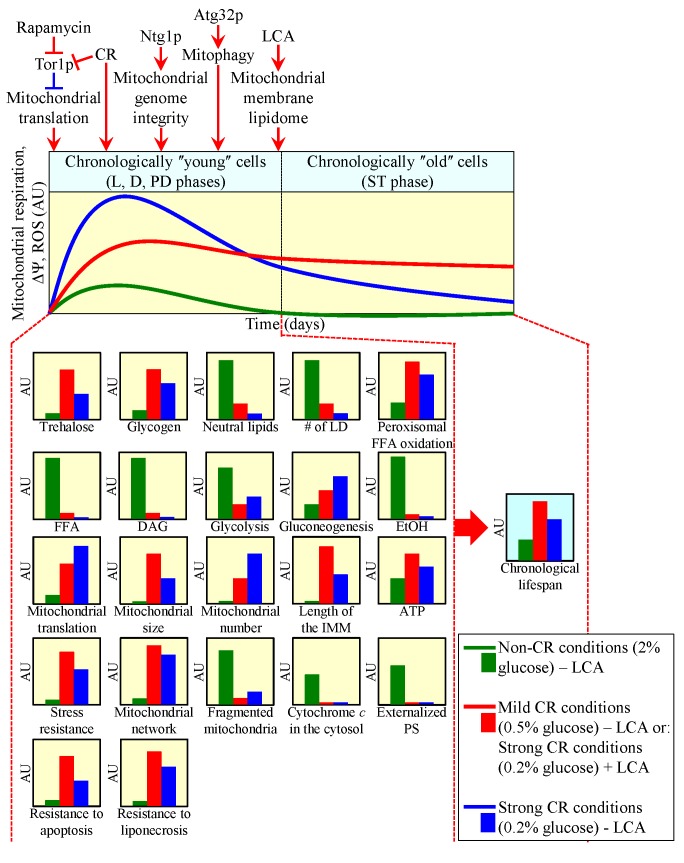
FIGURE 2: The functional state of mitochondria and mitochondrial ROS production in chronologically "young" yeast cells define their longevity by orchestrating numerous "downstream" cellular processes. A model for how coupled mitochondrial respiration, mitochondrial membrane potential maintenance and mitochondrial ROS production early in life of chronologically aging yeast cells, prior to entry into a non-proliferative state, define their viability after such entry - i.e., define their longevity. Activation arrows and inhibition bars denote pro-aging processes (displayed in blue color) or anti-aging processes (displayed in red color). Please see text for additional details. CR, caloric restriction; D, diauxic growth phase; DAG, diacylglycerol; EtOH, ethanol; FFA, non-esterified ("free") fatty acids; IMM, inner mitochondrial membrane; L, logarithmic growth phase; LCA, lithocholic acid; LD, lipid droplet; PD, post-diauxic growth phase; PS, phosphatidylserine; ROS, reactive oxygen species; ST, stationary growth phase; ΔΨ_m_, electrochemical potential across the IMM.

Recent studies revealed how various genetic, dietary and pharmacological interventions having diverse effects on the ETC, OXPHOS system and/or ROS production in mitochondria of chronologically "young" yeast impact yeast longevity 3,14,23,31,49,50,51,53,55,56,62,64,68,69,70,71,72,73,105,106,107,108. These studies suggest a model for how coupled mitochondrial respiration, mitochondrial membrane potential maintenance and mitochondrial ROS production early in life of chronologically aging yeast cells define their longevity. This model is depicted schematically in Figure 2. The model envisions that chronologically "young" yeast cells cultured under non-CR conditions develop and maintain a pro-aging cellular pattern because the capacities of these mitochondrial processes in such cells are below a certain level (Figure 2; these capacities are displayed in green color) 23,53,62,72,73. Furthermore, the model posits that chronologically "young" yeast cells, in which the capacities of these mitochondrial processes exceed a critical threshold, develop and maintain an anti-aging cellular pattern. This is because such capacities have specific impacts on: (1) some longevity-defining processes confined to mitochondria; and (2) certain longevity-defining processes in other cellular locations (Figure 2; these capacities are displayed in red color) 14,23,50,51,56,62,64,70,73,107. The model also predicts that a significant further increase in the capacities of coupled mitochondrial respiration, mitochondrial membrane potential maintenance and mitochondrial ROS production above a critical threshold in chronologically "young" yeast cells has a negative impact on their longevity (Figure 2; these capacities are displayed in blue color) 14,23,64,107.

The capacities of coupled mitochondrial respiration, mitochondrial membrane potential maintenance and mitochondrial ROS production in chronologically "young" yeast cells are modulated by several "upstream" pathways (Figure 2). These longevity-defining pathways include: (1) the nutrient- and energy-sensing TOR (target of rapamycin) signaling pathway, which through the rapamycin-sensitive protein kinase Tor1p inhibits mitochondrial translation of the OXPHOS enzymes encoded by mitochondrial DNA (mtDNA) 14,50,51,56,62; (2) a CR pathway, whose ability to modulate coupled mitochondrial respiration, mitochondrial membrane potential maintenance and mitochondrial ROS production in chronologically "young" yeast cells is mediated in part by Tor1p 3,14,23,33,50,51,52,56,62; (3) a pathway for the maintenance of mitochondrial genome integrity and copy number, which is orchestrated by the mitochondrial base-excision repair enzyme Ntg1p 108; (4) the mitophagy pathway of mitochondrial quality control responsible for autophagic degradation of aged, dysfunctional or damaged mitochondria - which requires the receptor protein Atg32p on the surface of mitochondria destined for such degradation 70; and (5) a pathway for specific remodeling of the membrane lipidome of mitochondria - which is stimulated in response to accumulation of the exogenously added lithocholic acid (LCA), an anti-aging natural compound, predominantly in the IMM 64,107 (Figure 2). Noteworthy, some of these "upstream" pathways overlap; such convergent pathways modulating the capacities of coupled mitochondrial respiration, mitochondrial membrane potential maintenance and mitochondrial ROS production in chronologically "young" yeast include the TOR and CR pathways 3,14,23,33,50,51,52,56,62, as well as the TOR and Ntg1p-governed pathways 108. In contrast, it seems that other "upstream" pathways modulating the capacities of these three longevity-defining mitochondrial processes do not converge and act in synergy; such "parallel" pathways include: (1) a CR pathway and an LCA-driven pathway for remodeling of the membrane lipidome of mitochondria 64,107; and (2) a CR pathway and the Ntg1p-governed pathway 108.

The capacities of coupled mitochondrial respiration, mitochondrial membrane potential maintenance and mitochondrial ROS production in chronologically "young" yeast cells define their longevity by orchestrating numerous "downstream" cellular processes throughout lifespan - i.e., prior to entry into a non-proliferative state and after such entry (Figure 2). Among them are the following "downstream" processes (Figure 2): (1) the maintenance of trehalose homeostasis, a longevity-defining process known to modulate proteostasis in chronologically "young" and "old" cells 23,61 (Figure 1A); (2) the maintenance of the homeostasis of glycogen, a reserve carbohydrate whose elevated level in chronologically "young" and "old" cells is a hallmark of carbohydrate metabolism remodeling in yeast cultured under CR 23; (3) the maintenance of the homeostasis of neutral lipids deposited within LD, a process known to play an essential role in regulating longevity of chronologically aging yeast 14,23,92 (Figure 1B); (4) peroxisomal oxidation of fatty acids, a process implicated in yeast chronological aging 14,23,91,92 (Figure 1B); (5) the maintenance of the homeostasis of non-esterified ("free") fatty acids and diacylglycerol, whose reduced levels in chronologically "young" and "old" cells are characteristic of lipid metabolism remodeling in yeast cultured under CR - a pattern likely linked to the demonstrated abilities of both lipid species to elicit an age-related form of liponecrotic PCD 14,23,103 (Figure 1B); (6) the maintenance of a balance between the relative rates of glycolysis and gluconeogenesis, a process known to impact the level of ethanol in chronologically "young" and "old" yeast - thus defining the extent to which this product of glucose fermentation suppresses the longevity-extending process of peroxisomal oxidation of fatty acids 14,23 (Figure 1B); (7) the longevity-extending process of mitochondrial translation 23,50,51,56; (8) the maintenance of a balance between the relative rates of mitochondrial fusion and fission, a process known to define the size and number of mitochondria, the length of mitochondrial cristae extending from the IMM, and the level of ATP synthesized in mitochondria of chronologically "young" and "old" yeast 14,23,64,70; (9) the development of an age-related pattern of susceptibility to chronic oxidative, thermal and osmotic stresses 3,14,23,33,50,56,59,62; (10) an age-related form of apoptotic PCD, which in chronologically "young" yeast is manifested in such early hallmark events of this PCD as the fragmentation of a tubular mitochondrial network into individual mitochondria, release of cytochrome c from mitochondria into the cytosol and phosphatidylserine (PS) translocation from the inner to the outer leaflet of the plasma membrane 14,23 (Figure 1B); and (11) the development of a pattern of cell susceptibility to an age-related forms of apoptotic and liponecrotic PCD elicited by an exposure to exogenous hydrogen peroxide or palmitoleic acid, respectively 14,23,59,103.

The molecular mechanisms through which the capacity of mitochondrial ROS production in chronologically "young" yeast cells defines their longevity have begun to emerge; they involve communication between mitochondria and the nucleus via two signaling pathways 71,108. In one of these signaling pathways, hormetic concentrations of ROS released from mitochondria trigger a pro-longevity transcriptional program in the nucleus by stimulating Gis1p, Msn2p and Msn4p 68,71,108; these transcriptional factors are known to activate expression of numerous genes essential for the resistance to various stresses, stationary phase survival, carbohydrate metabolism, nutrient sensing and chronological longevity assurance 109,110,111. Another mitochondria-to-nucleus signaling pathway initiated by hormetic concentrations of ROS in chronologically "young" yeast cells involves a cascade of events within the nucleus. In this cascade, the DNA damage response (DDR) kinase Tel1p responds to hormetic concentrations of ROS released from mitochondria by phosphorylating and activating the DDR kinase Rad53p. Active Rad53p then phosphorylates and inactivates the histone demethylase Rph1p confined to subtelomeric chromatin regions, thereby repressing their transcription, minimizing telomeric DNA damage and ultimately extending longevity of chronologically aging yeast 68,71. Noteworthy, it seems that the Tel1p-Rad53p-Rph1p signaling pathway overlaps with and is regulated by the "upstream" Ntg1p-governed pathway (Figure 2) for the maintenance of mitochondrial genome integrity and copy number 108.

Altogether, these findings suggest that there is a checkpoint early in life of chronologically aging yeast cells (i.e., prior to entry into a non-proliferative state) at which the capacities of coupled mitochondrial respiration, mitochondrial membrane potential maintenance and mitochondrial ROS production define their viability after such entry - i.e., define their longevity. It seems that the capacities of these three mitochondrial processes at such checkpoint define yeast longevity by orchestrating a number of "downstream" processes taking place in various cellular locations throughout lifespan, before an arrest of cell growth and division, and following such arrest.

### Metabolite flow within glycolytic and non-glycolytic pathways of carbohydrate metabolism defines the establishment of a pro- or anti-aging cellular pattern

**Figure 3:**
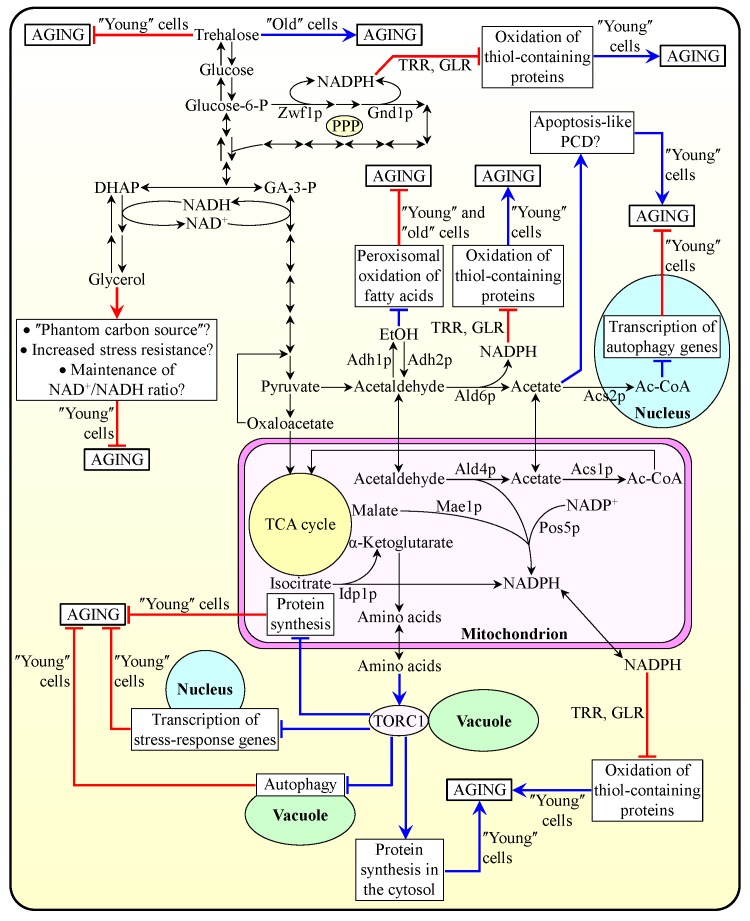
FIGURE 3: The coordinated metabolite flow within glycolytic and non-glycolytic pathways of carbohydrate metabolism defines yeast longevity by modulating vital cellular processes. A model for how metabolic flux within the network integrating glycolytic and non-glycolytic pathways of carbohydrate metabolism in chronologically "young", proliferating yeast cells define the development and maintenance of a pro- or anti-aging cellular pattern throughout lifespan, before an arrest of cell growth and division and after such arrest. Activation arrows and inhibition bars denote pro-aging processes (displayed in blue color) or anti-aging processes (displayed in red color). Please see text for additional details. Ac-CoA, acetyl-CoA; DHAP, dihydroxyacetone phosphate; EtOH, ethanol; GA-3-P, glyceraldehyde-3-phosphate; GLR, glutathione reductase; PCD, programmed cell death; TCA, tricarboxylic acid; TORC1, target of rapamycin complex 1; TRR, thioredoxin reductase.

Recent studies provided evidence that the relative rates of reactions comprising glycolytic and non-glycolytic pathways of carbohydrate metabolism, as well as the intracellular concentrations of some key intermediates in these pathways, define the development and maintenance of a pro- or anti-aging cellular pattern in chronologically aging yeast 3,10,23,33,52,54,57,58,63,65,66,67,69,84,112,113,114,115,116,117. The major findings of these studies can be summarized as follows: (1) the establishment of a certain metabolic pattern of such coordinated pathways early in life of chronologically aging yeast cells, prior to entry into a non-proliferative state, defines their longevity; and (2) some dietary, genetic and pharmacological interventions extend yeast longevity by causing a specific remodeling of such metabolic pathways in chronologically "young", proliferating cells 23,52,66,67,69,112,113,114,115,116,117. These findings suggest a model for how metabolic flux within the network integrating glycolytic and non-glycolytic pathways of carbohydrate metabolism modulates longevity-defining cellular processes. This model is depicted schematically in Figure 3.

The model posits that glucose, the primary carbon source used in most assays for investigating yeast chronological aging under laboratory conditions, is initially converted to pyruvate via the glycolytic pathway and also enters the pentose phosphate pathway (PPP) 23,25,27,30,33,57,66 (Figure 3). In chronologically "young" yeast cells progressing through logarithmic (L) phase, the PPP generates not only ribose-5-phosphate for nucleic acid synthesis but also NADPH, the primary source of cellular reducing equivalents required for the reductive synthesis of fatty acids, sterols and some amino acids 60,63,66,118. Importantly, NADPH - which is produced in the Zwf1p- and Gnd1p-driven oxidative reactions of the PPP - also functions as the electron donor essential for sustaining cellular redox homeostasis via thioredoxin and glutathione reductase systems 63,66,118 (Figure 3). These two NADPH-dependent systems have been shown to reduce the extent of oxidative damage to numerous thiol-containing cytosolic, nuclear and mitochondrial proteins in chronologically "young" yeast cells; as such, both reductase systems play essential roles in longevity assurance and underlie, in part, the robust longevity-extending effect of CR 63,66 (Figure 3).

After chronologically aging yeast cells consume glucose during L phase, they enter diauxic (D) and then post-diauxic (PD) phases of slow growth 23,25,30,33. During D and PD phases, prior to an arrest of cell growth and division and entry into the non-proliferative stationary (ST) phase, the pyruvate formed by glycolysis can enter several alternative pathways of carbon metabolism; all these pathways have been implicated in modulating various longevity-defining cellular processes 3,10,23,33,52,54,57,58,63,65,66,67,69,84,112,113,114,115,116,117. One of these alternative metabolic pathways is fermentation leading to the formation of ethanol and/or acetic acid in the cytosol of a chronologically "young" yeast cell; the nature of a product of such fermentation depends on the type of a synthetic or nutrient-rich growth medium used and/or aeration conditions applied 3,10,23,33,52,112,113,114,115,117 (Figure 3). As discussed above, the level of ethanol in chronologically aging yeast defines the extent to which it suppresses the longevity-extending process of peroxisomal oxidation of fatty acids 14,23 (Figure 1B). The steady-state level of this product of glucose fermentation depends on the relative enzymatic activities of Adh1p and Adh2p, which are required for ethanol formation or oxidation, respectively 3,23,52,112,117 (Figure 3). Acetic acid, the alternative product of glucose fermentation in the cytosol of chronologically "young" yeast cells, shortens their longevity - likely because it can elicit an age-related form of apoptotic PCD 3,27,113,114,115 (Figure 3). Of note, the Ald6p-dependent acetaldehyde dehydrogenase reaction in the cytosol of these cells yields not only acetic acid but also NADPH 60,66,118 (Figure 3). As discussed above, the NADPH-dependent thioredoxin and glutathione reductase systems are vital for longevity assurance and are essential for the longevity-extending effect of CR because they reduce the extent of oxidative damage to many thiol-containing cellular proteins in chronologically "young" yeast cells 63,66,118 (Figure 3). Moreover, acetic acid can be converted to acetyl-CoA in the nucleo-cytosolic Acs2p-dependent reaction 116,118. The acetyl-CoA formed in the nucleus has been shown to shorten longevity of chronologically aging yeast by causing histone H3 hyperacetylation, thereby selectively suppressing transcription of the *ATG5*,* ATG7*,* ATG11 *and* ATG14* genes; these genes encode proteins needed for the longevity-extending process of autophagy 116 (Figure 3). It remains to be determine what are the relative impacts of acetic acid (a pro-aging metabolite), NADPH (an anti-aging metabolite) and acetyl-CoA (a pro-aging metabolite) on longevity of chronologically aging yeast.

The model depicted in Figure 3 further envisions that glucose fermentation to glycerol in the cytosol of a chronologically "young" yeast cell operates as a longevity-extending cellular process - likely because it reduces metabolite flow into the longevity-shortening cellular process of glucose fermentation to ethanol and/or acetic acid 52,113 (Figure 3). The term "phantom carbon source" has been coined for defining this aspect of the essential longevity-extending role of glycerol in yeast 52. Glycerol formed by glucose fermentation in the cytosol of a chronologically "young" yeast cell has been proposed to extend it lifespan also because it is known to reduce cell susceptibility to chronic oxidative, thermal and osmotic stresses 52 (Figure 3). Furthermore, glucose fermentation to glycerol in chronologically "young" yeast cells may also play an essential role in longevity assurance by maintaining an NAD^+^/NADH ratio characteristic of an anti-aging cellular pattern 52 (Figure 3).

In addition to being converted to ethanol, acetic acid, NADPH and/or acetyl-CoA, the pyruvate formed by glycolysis can enter the gluconeogenesis pathway leading to the formation of glucose; in chronologically aging yeast, this newly synthesized glucose can be further used for the synthesis of trehalose 23,61,62,117 (Figure 3). As discussed above 23,61 (Figure 1A): (1) in chronologically "young" yeast cells progressing through D and PD growth phases, trehalose plays an essential longevity-extending role, whereas (2) in chronologically "old", non-proliferating cells, trehalose plays a key role in shortening longevity (Figure 1).

In chronologically "young" yeast cells progressing through D and PD growth phases, the pyruvate formed by glycolysis - as well as the acetaldehyde and acetate derived from it - can also fuel several longevity-defining metabolic processes in mitochondria. One of these metabolic processes is the TCA cycle. Two intermediates of the cycle, malate and isocitrate, can be used to form NADPH in the Mae1p- and Idp1p-dependent reactions, respectively 60,66,118 (Figure 3). NADPH in mitochondria of chronologically "young" yeast cells progressing through D and PD growth phases can also be formed in the Ald4p-dependent acetaldehyde dehydrogenase reaction and in the Pos5p-dependent NADH kinase reaction 60,66,118 (Figure 3). As discussed above, NADPH can play a vital longevity-extending role by being used for a thioredoxin- and glutathione reductase-driven decrease in the extent of oxidative damage to thiol-containing mitochondrial proteins and proteins in other cellular locations 63,66,118 (Figure 3). Moreover, the oxaloacetate and α-ketoglutarate intermediates of the TCA cycle in mitochondria of chronologically "young" yeast cells progressing through D and PD growth phases are known to be used for the synthesis of amino acids 60,118,119 (Figure 3). After their exit from mitochondria to the cytosol, some of these amino acids - including aspartate, asparagine, glutamate and glutamine - cause a significant increase in protein kinase activity of the TOR complex 1 (TORC1) on the surface of vacuoles 20,119,120,121,122,123,124 (Figure 3). The resulting stimulation of the TOR signaling pathway in chronologically "young" yeast cells is known to initiate the establishment of a pro-aging cellular pattern by: (1) activating the longevity-shortening process of protein synthesis in the cytosol; (2) suppressing the longevity-extending process of autophagy in vacuoles; (3) inhibiting the longevity-extending process of transcription of numerous stress-response genes in the nucleus; and (4) suppressing the longevity-extending process of protein synthesis in mitochondria 20,50,51,119,120,121,122,123,124 (Figure 3).

Taken together, these findings strongly suggest that there are several checkpoints early in life of chronologically aging yeast cells - during L, D and PD phases preceding entry into the non-proliferative ST phase - at which the coordinated metabolite flow within glycolytic and non-glycolytic pathways of carbohydrate metabolism defines yeast longevity. It seems that at each of these early-life checkpoints some key intermediates in such pathways affect - in a different manner and in a concentration-dependent fashion - the vital processes of cell metabolism, growth, proliferation, stress resistance, macromolecular homeostasis, survival and death. By modulating such longevity-defining cellular processes throughout lifespan - prior to an arrest of cell growth and division and following such arrest - these key metabolic intermediates define the development and maintenance of a pro- or anti-aging cellular pattern.

## A STEPWISE PROGRESSION OF A BIOMOLECULAR NETWORK OF CELLULAR AGING THROUGH A SERIES OF LIFESPAN CHECKPOINTS DEFINES LONGEVITY OF CHRONOLOGICALLY AGING YEAST

**Figure 4:**
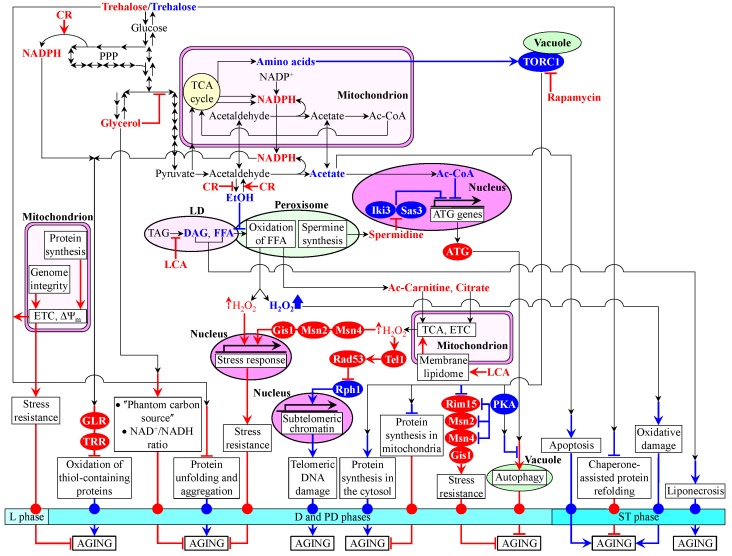
FIGURE 4: A concept of a biomolecular network underlying chronological aging in yeast. A model for how a stepwise progression of a biomolecular network of cellular aging through a series of lifespan checkpoints is monitored by some checkpoint-specific "master regulator" proteins. The model posits that a synergistic action of these master regulator proteins at several early-life and late-life checkpoints modulates certain vital cellular processes throughout lifespan - thereby orchestrating the development and maintenance of a pro- or anti-aging cellular pattern and, thus, defining longevity of chronologically aging yeast. Activation arrows and inhibition bars denote pro-aging processes (displayed in blue color) or anti-aging processes (displayed in red color). Pro-aging or anti-aging master regulator proteins are displayed in blue color or red color, respectively. Ac-Carnitine, acetyl-carnitine; Ac-CoA, acetyl-CoA; CR, caloric restriction; D, diauxic growth phase; DAG, diacylglycerol; ETC, electron transport chain; EtOH, ethanol; FFA, non-esterified ("free") fatty acids; GLR, glutathione reductase; L, logarithmic growth phase; LCA, lithocholic acid; LD, lipid droplet; PD, post-diauxic growth phase; PKA, protein kinase A; TAG, triacylglycerols; TCA, tricarboxylic acid cycle; ST, stationary growth phase; TORC1, target of rapamycin complex 1; TRR, thioredoxin reductase; ΔΨ_m_, electrochemical potential across the inner mitochondrial membrane.

The above analysis of the current knowledge about cell-autonomous mechanisms underlying chronological aging in yeast suggests the existence of several lifespan checkpoints that are critically important for establishing the pace of such aging. A recently reported ability of a natural chemical compound to extend longevity of chronologically aging yeast only if added at some of these checkpoints 59 supports the notion that a stepwise progression through such checkpoints may define the development and maintenance of a pro- or anti-aging cellular pattern. Therefore, in this review we extend the recently proposed network theories of yeast chronological aging 3,20,23,58,59,61,62,125,126,127 by putting forward a concept of a biomolecular network underlying the chronology of cellular aging in yeast. This concept is depicted schematically in Figure 4. The concept posits that the network progresses through a series of the early-life checkpoints (that exist in L, D and PD phases) and late-life checkpoints (that exist in ST phase). At each of these checkpoints, the intracellular concentrations of some key intermediates and products of certain metabolic pathways - as well as the rates of coordinated flow of such metabolites within an intricate network of intercompartmental (i.e., organelle-organelle and organelle-cytosol) communications - are monitored by some checkpoint-specific "master regulator" proteins (Figure 4). The concept further envisions that, because each of these master regulator proteins is known for its essential role in longevity regulation, their synergistic action at certain early-life and late-life checkpoints modulates the rates and efficiencies of progression of such essential processes as cell metabolism, growth, proliferation, stress resistance, macromolecular homeostasis, survival and death (Figure 4). The concept predicts that, by modulating these vital cellular processes throughout lifespan - prior to an arrest of cell growth and division and following such arrest - the checkpoint-specific master regulator proteins orchestrate the development and maintenance of a pro- or anti-aging cellular pattern and, thus, define longevity of chronologically aging yeast (Figure 4).

In the proposed concept of a biomolecular network underlying the chronology of cellular aging in yeast, the key intermediates and products of longevity-defining metabolic pathways include the following metabolites: NADPH (an anti-aging metabolite), glycerol (an anti-aging metabolite), trehalose (an anti-aging metabolite in "young" cells but a pro-aging metabolite in "old" cells), acetyl-carnitine (an anti-aging metabolite), citrate (an anti-aging metabolite), hydrogen peroxide (an anti-aging metabolite if it is maintained at an "adaptive" level eliciting a hormetic response but a pro-aging metabolite if it amasses above a cytotoxic level), spermidine (an anti-aging metabolite), ethanol (a pro-aging metabolite), acetic acid (a pro-aging metabolite), acetyl-CoA (a pro-aging metabolite), non-esterified fatty acids (pro-aging metabolites), diacylglycerol (a pro-aging metabolite) and amino acids (pro-aging metabolites) (Figure 4). The proposed concept posits that these key metabolites undergo an age-related flow within an intricate network of intercompartmental communications. Such unidirectional and bidirectional flow of the critical metabolites between different cellular compartments connects mitochondria and the nucleus, peroxisomes and the nucleus, vacuoles and the nucleus, the cytosol and the nucleus, mitochondria and peroxisomes, lipid droplets and peroxisomes, mitochondria and vacuoles, peroxisomes and the cytosol, mitochondria and the cytosol, and vacuoles and the cytosol (Figure 4). In the proposed concept, a CR diet and some pharmacological interventions (such as rapamycin and LCA) - as well as some other environmental cues (such as the intake of certain dietary supplements and hormetic environmental stresses) - extend longevity of chronologically aging yeast by altering the age-related dynamics of changes in the intracellular concentrations of the key metabolites and/or by modulating the intercompartmental flow of these critical metabolites (Figure 4). The following proteins and protein complexes operate as checkpoint-specific master regulators that - according to the proposed concept - respond to age-related changes in the intracellular concentrations of the key metabolites and in the intensity of their intercompartmental flow by modulating the longevity-defining processes of cell metabolism, growth, proliferation, stress resistance, macromolecular homeostasis, survival and death: (1) thioredoxin and glutathione reductase systems (anti-aging master regulators); (2) Rim15p, Msn2p, Msn4p and Gis1p (anti-aging master regulators); (3) Tel1p and Rad53p (anti-aging master regulators); (4) components of the ATG protein machinery involved in the essential cytoprotective cellular process of autophagy (anti-aging master regulators); (5) Rph1p (a pro-aging master regulator); (6) Iki3p and Sas3p (pro-aging master regulators); and (7) TORC1 and protein kinase A (PKA) (pro-aging master regulators) (Figure 4). Future studies are likely to reveal some other checkpoint-specific master regulator proteins. Finally, the proposed concept posits that the checkpoint-specific master regulators define longevity of chronologically aging yeast by orchestrating certain "downstream" cellular processes and features throughout lifespan - i.e., prior to entry into a non-proliferative state and after such entry (Figure 4). These "downstream" processes and features include (Figure 4): (1) susceptibility to chronic oxidative, thermal and osmotic stresses; (2) oxidative damage to numerous thiol-containing cytosolic, nuclear and mitochondrial proteins; (3) cellular proteostasis, which depends on the relative rates and efficiencies of protein folding, misfolding, unfolding, refolding, oxidative damage, solubility and aggregation; (4) transcription of subtelomeric chromatin regions, which defines the extent of age-related telomeric DNA damage; (5) protein synthesis in the cytosol; (6) protein synthesis in mitochondria; (7) autophagy, an essential mechanism of cellular quality control responsible for the autophagic degradation of aged, dysfunctional and damaged macromolecules and organelles; and (8) liponecrotic and apoptotic forms of age-related PCD.

## CONCLUSIONS 

Emergent evidence supports the view that the processes of cell metabolism, growth, proliferation, stress resistance, macromolecular homeostasis, survival and death in chronologically aging yeast are integrated into a biomolecular network of cellular aging. Recent findings imply that a stepwise progression of this network through a series of the early-life and late-life checkpoints is monitored by some checkpoint-specific master regulator proteins; these proteins act in synergy to orchestrate the development and maintenance of a pro- or anti-aging cellular pattern and, thus, to define longevity of chronologically aging yeast. The major challenge now is to understand if a process of yeast chronological aging - whose progression through a series of lifespan checkpoints is monitored and controlled by a distinct set of master regulator proteins - is a program. We hypothesize that, although yeast chronological aging is a gradual and controllable process progressing through a series of consecutive lifespan checkpoints, it is not a program aimed at the termination of lives of individual yeast cells and of their organized populations. Rather, chronological aging in yeast is probably due to the inability of chronologically "young", proliferating cells to maintain the capacities of some crucial cellular processes above a critical threshold; these key cellular processes may include ones that ensure robust cell growth and proliferation, implement a spatiotemporal control of cell development and differentiation into quiescent and non-quiescent cell populations, and limit an age-related accumulation of molecular and cellular damage. The proposed here hypothesis also posits that the extreme cellular stress caused by the excessive accumulation of such damage in chronologically "old", non-proliferating cells may activate pathways known to orchestrate the apoptotic, regulated necrotic, autophagic and/or liponecrotic subroutines of PCD; these subroutines are believed to constitute modules that are dynamically integrated into a so-called PCD network 103,128,129,130,131,132,133,134,135. In sum, the hypothesis of non-programmed chronological aging in yeast envisions that: (1) the processes of cell metabolism, growth, proliferation, stress response, macromolecular homeostasis, development and differentiation have evolved in the course of natural selection within diverse ecosystems (and perhaps under laboratory conditions) as programs aimed at sustaining the long-term survival of individual yeast cells under various environmental conditions; (2) the processes of various subroutines of PCD have evolved throughout natural selection within different ecosystems (and perhaps under laboratory conditions) as "altruistic" programs aimed at sustaining the long-term survival of organized yeast populations (such as colonies and biofilms of yeast cells attached to solid surfaces) by eliminating individual yeast cells that are damaged, unable to mate and reproduce, poorly adapted to diverse environmental conditions, and/or release excessive quantities of ROS and other harmful metabolites (for a comprehensive discussion of this topic, see refs. 136,137); and (3) a trade-off between these programs aimed at sustaining the long-term survival of individual yeast cells or that of organized yeast populations under diverse environmental conditions drives the evolution of yeast longevity towards maintaining a finite yeast chronological lifespan within an ecosystem. The proposed here hypothesis of non-programmed chronological aging in yeast provides a framework for future studies aimed at testing its validity.

## References

[1] Fontana L, Partridge L, Longo VD (2010). Extending healthy life span - from yeast to
humans.. Science.

[2] Kaeberlein M (2010). Lessons on longevity from budding yeast.. Nature.

[3] Longo VD, Shadel GS, Kaeberlein M, Kennedy B (2012). Replicative and chronological aging in Saccharomyces
cerevisiae.. Cell Metab.

[4] Váchová L, Cáp M, Palková Z (2012). Yeast colonies: a model for studies of aging, environmental
adaptation, and longevity.. Oxid Med Cell Longev.

[5] Denoth Lippuner A, Julou T, Barral Y (2014). Budding yeast as a model organism to study the effects of
age.. FEMS Microbiol Rev.

[6] Nyström T, Liu B (2014). Protein quality control in time and space - links to cellular
aging.. FEMS Yeast Res.

[7] Weissman J, Guthrie C, Fink GR (2010). Guide to Yeast Genetics: Functional Genomics, Proteomics, and
Other Systems Analysis.. Acamdemic Press, Burlington.

[8] Botstein D, Fink GR (2011). Yeast: an experimental organism for 21st Century
biology.. Genetics.

[9] Lee SS, Avalos Vizcarra I, Huberts DH, Lee LP, Heinemann M (2012). Whole lifespan microscopic observation of budding yeast aging
through a microfluidic dissection platform.. Proc Natl Acad Sci USA.

[10] Sutphin GL, Olsen BA, Kennedy BK, Kaeberlein M (2012). Genome-wide analysis of yeast aging.. Subcell Biochem.

[11] Xie Z, Zhang Y, Zou K, Brandman O, Luo C, Ouyang Q, Li H (2012). Molecular phenotyping of aging in single yeast cells using a
novel microfluidic device.. Aging Cell.

[12] Zhang Y, Luo C, Zou K, Xie Z, Brandman O, Ouyang Q, Li H (2012). Single cell analysis of yeast replicative aging using a new
generation of microfluidic device.. PLoS One.

[13] Eisenberg T, Knauer H, Schauer A, Büttner S, Ruckenstuhl C, Carmona-Gutierrez D, Ring J, Schroeder S, Magnes C, Antonacci L, Fussi H, Deszcz L, Hartl R, Schraml E, Criollo A, Megalou E, Weiskopf D, Laun P, Heeren G, Breitenbach M, Grubeck-Loebenstein B, Herker E, Fahrenkrog B, Fröhlich KU, Sinner F, Tavernarakis N, Minois N, Kroemer G, Madeo F (2009). Induction of autophagy by spermidine promotes
longevity.. Nat Cell Biol.

[14] Goldberg AA, Richard VR, Kyryakow P, Bourque SD, Beach A, Burstein MT, Glebov A, Koupaki O, Boukh-Viner T, Gregg C, Juneau M, English AM, Thomas DY, Titorenko VI (2010a). Chemical genetic screen identifies lithocholic acid as an
anti-aging compound that extends yeast chronological life span in a
TOR-independent manner, by modulating housekeeping longevity assurance
processes.. Aging.

[15] Kapahi P, Chen D, Rogers AN, Katewa SD, Li PW, Thomas EL, Kockel L (2010). With TOR, less is more: a key role for the conserved
nutrient-sensing TOR pathway in aging.. Cell Metab.

[16] Evans DS, Kapahi P, Hsueh WC, Kockel L (2011). TOR signaling never gets old: aging, longevity and TORC1
activity.. Ageing Res Rev.

[17] Minois N, Carmona-Gutierrez D, Madeo F (2011). Polyamines in aging and disease.. Aging.

[18] Jazwinski SM (2012). The retrograde response and other pathways of interorganelle
communication in yeast replicative aging.. Subcell Biochem.

[19] Jazwinski SM (2013). The retrograde response: when mitochondrial quality control is
not enough.. Biochim Biophys Acta.

[20] Leonov A, Titorenko VI (2013). A network of interorganellar communications underlies cellular
aging.. IUBMB Life.

[21] Hubbard BP, Sinclair DA (2014). Small molecule SIRT1 activators for the treatment of aging and
age-related diseases.. Trends Pharmacol Sci.

[22] Sinclair DA, Guarente L (2014). Small-molecule allosteric activators of sirtuins.. Annu Rev Pharmacol Toxicol.

[23] Goldberg AA, Bourque SD, Kyryakov P, Gregg C, Boukh-Viner T, Beach A, Burstein MT, Machkalyan G, Richard V, Rampersad S, Cyr D, Milijevic S, Titorenko VI (2009a). Effect of calorie restriction on the metabolic history of
chronologically aging yeast.. Exp Gerontol.

[24] Steffen KK, Kennedy BK, Kaeberlein M (2009). Measuring replicative life span in the budding
yeast.. J Vis Exp.

[25] Hu J, Wei M, Mirisola MG, Longo VD (2013). Assessing chronological aging in Saccharomyces
cerevisiae.. Methods Mol Biol.

[26] Sinclair DA (2013). Studying the replicative life span of yeast
cells.. Methods Mol Biol.

[27] Burtner CR, Murakami CJ, Kaeberlein M (2009). A genomic approach to yeast chronological aging.. Methods Mol Biol.

[28] Murakami C, Kaeberlein M (2009). Quantifying yeast chronological life span by outgrowth of aged
cells.. J Vis Exp.

[29] Wu Z, Song L, Liu SQ, Huang D (2011). A high throughput screening assay for determination of
chronological lifespan of yeast.. Exp Gerontol.

[30] Fabrizio P, Longo VD (2007). The chronological life span of Saccharomyces
cerevisiae.. Methods Mol Biol.

[31] Piper PW (2012). Maximising the yeast chronological lifespan.. Subcell Biochem.

[32] Longo VD, Kennedy BK (2006). Sirtuins in aging and age-related disease.. Cell.

[33] Longo VD, Fabrizio P (2012). Chronological aging in Saccharomyces cerevisiae.. Subcell Biochem.

[34] Bitterman KJ, Medvedik O, Sinclair DA (2003). Longevity regulation in Saccharomyces cerevisiae: linking
metabolism, genome stability, and heterochromatin.. Microbiol Mol Biol Rev.

[35] Steinkraus KA, Kaeberlein M, Kennedy BK (2008). Replicative aging in yeast: the means to the end.. Annu Rev Cell Dev Biol.

[36] St'ovíček V, Váchová L, Kuthan M, Palková Z (2010). General factors important for the formation of structured
biofilm-like yeast colonies.. Fungal Genet Biol.

[37] Váchová L, Palková Z (2011). Aging and longevity of yeast colony populations: metabolic
adaptation and differentiation.. Biochem Soc Trans.

[38] Cáp M,  Stěpánek  L, Harant K,  Váchová  L,  Palková Z (2012a). Cell differentiation within a yeast colony: metabolic and
regulatory parallels with a tumor-affected organism.. Mol Cell.

[39] Cáp M,  Váchová  L,  Palková  Z (2012b). Reactive oxygen species in the signaling and adaptation of
multicellular microbial communities.. Oxid Med Cell Longev.

[40] Mazzoni C, Mangiapelo E, Palermo V, Falcone C (2012). Hypothesis: is yeast a clock model to study the onset of humans
aging phenotypes?. Front Oncol.

[41] Váchová L, Hatáková L, Cáp M, Pokorná M, Palková Z (2013). Rapidly developing yeast microcolonies differentiate in a similar
way to aging giant colonies.. Oxid Med Cell Longev.

[42] Palková Z, Wilkinson D, Váchová L (2014). Aging and differentiation in yeast populations: Elders with
different properties and functions.. FEMS Yeast Res.

[43] Sťovíček V, Váchová L, Begany M, Wilkinson D, Palková Z (2014). Global changes in gene expression associated with phenotypic
switching of wild yeast.. BMC Genomics.

[44] Howitz KT, Sinclair DA (2008). Xenohormesis: sensing the chemical cues of other
species.. Cell.

[45] Goldberg AA, Kyryakov P, Bourque SD, Titorenko VI (2010b). Xenohormetic, hormetic and cytostatic selective forces driving
longevity at the ecosystemic level.. Aging.

[46] Burstein MT, Beach A, Richard VR, Koupaki O, Gomez-Perez A, Goldberg AA, Kyryakov P, Bourque SD, Glebov A, Titorenko VI (2012a). Interspecies chemical signals released into the environment may
create xenohormetic, hormetic and cytostatic selective forces that drive the
ecosystemic evolution of longevity regulation mechanisms.. Dose Response.

[47] Howitz KT, Bitterman KJ, Cohen HY, Lamming DW, Lavu S, Wood JG, Zipkin RE, Chung P, Kisielewski A, Zhang LL, Scherer B, Sinclair DA (2003). Small molecule activators of sirtuins extend Saccharomyces
cerevisiae lifespan.. Nature.

[48] Lamming DW, Wood JG, Sinclair DA (2004). Small molecules that regulate lifespan: evidence for
xenohormesis.. Mol Microbiol.

[49] Bonawitz ND, Shadel GS (2007). Rethinking the mitochondrial theory of aging: the role of
mitochondrial gene expression in lifespan determination.. Cell Cycle.

[50] Bonawitz ND, Chatenay-Lapointe M, Pan Y, Shadel GS (2007). Reduced TOR signaling extends chronological life span via
increased respiration and upregulation of mitochondrial gene
expression.. Cell Metab.

[51] Pan Y, Shadel GS (2009). Extension of chronological life span by reduced TOR signaling
requires down-regulation of Sch9p and involves increased mitochondrial
OXPHOS complex density.. Aging.

[52] Wei M, Fabrizio P, Madia F, Hu J, Ge H, Li LM, Longo VD (2009). Tor1/Sch9-regulated carbon source substitution is as effective as
calorie restriction in life span extension.. PLoS Genet.

[53] Mesquita A, Weinberger M, Silva A, Sampaio-Marques B, Almeida B, Leão C, Costa V, Rodrigues F, Burhans WC, Ludovico P (2010). Caloric restriction or catalase inactivation extends yeast
chronological lifespan by inducing H2O2 and superoxide dismutase
activity.. Proc Natl Acad Sci USA.

[54] Beach A, Titorenko VI (2011). In search of housekeeping pathways that regulate
longevity.. Cell Cycle.

[55] Pan Y (2011). Mitochondria, reactive oxygen species, and chronological aging: a
message from yeast.. Exp Gerontol.

[56] Pan Y, Schroeder EA, Ocampo A, Barrientos A, Shadel GS (2011). Regulation of yeast chronological life span by TORC1 via adaptive
mitochondrial ROS signaling.. Cell Metab.

[57] Titorenko VI, Terlecky SR (2011). Peroxisome metabolism and cellular aging.. Traffic.

[58] Beach A, Burstein MT, Richard VR, Leonov A, Levy S, Titorenko VI (2012). Integration of peroxisomes into an endomembrane system that
governs cellular aging.. Front Physiol.

[59] Burstein MT, Kyryakov P, Beach A, Richard VR, Koupaki O, Gomez-Perez A, Leonov A, Levy S, Noohi F, Titorenko VI (2012b). Lithocholic acid extends longevity of chronologically aging
yeast only if added at certain critical periods of their
lifespan.. Front Physiol.

[60] Cai L, Tu BP (2012). Driving the cell cycle through metabolism.. Annu Rev Cell Dev Biol.

[61] Kyryakov P, Beach A, Richard VR, Burstein MT, Leonov A, Levy S, Titorenko VI (2012). Caloric restriction extends yeast chronological lifespan by
altering a pattern of age-related changes in trehalose
concentration.. Front Physiol.

[62] Ocampo A, Liu J, Schroeder EA, Shadel GS, Barrientos A (2012). Mitochondrial respiratory thresholds regulate yeast chronological
life span and its extension by caloric restriction.. Cell Metab.

[63] Barral Y (2013). A new answer to old questions.. eLife.

[64] Beach A, Richard VR, Leonov A, Burstein MT, Bourque SD, Koupaki O, Juneau M, Feldman R, Iouk T, Titorenko VI (2013). Mitochondrial membrane lipidome defines yeast
longevity.. Aging.

[65] Beach A, Titorenko VI (2013). Essential roles of peroxisomally produced and metabolized
biomolecules in regulating yeast longevity.. Subcell Biochem.

[66] Brandes N, Tienson H, Lindemann A, Vitvitsky V, Reichmann D, Banerjee R, Jakob U (2013). Time line of redox events in aging postmitotic
cells.. eLife.

[67] Hachinohe M, Yamane M, Akazawa D, Ohsawa K, Ohno M, Terashita Y, Masumoto H (2013). A reduction in age-enhanced gluconeogenesis extends
lifespan.. PLoS One.

[68] Mirisola MG, Longo VD (2013). A radical signal activates the epigenetic regulation of
longevity.. Cell Metab.

[69] Orlandi I, Ronzulli R, Casatta N, Vai M (2013). Ethanol and acetate acting as carbon/energy sources negatively
affect yeast chronological aging.. Oxid Med Cell Longev.

[70] Richard VR, Leonov A, Beach A, Burstein MT, Koupaki O, Gomez-Perez A, Levy S, Pluska L, Mattie S, Rafesh R, Iouk T, Sheibani S, Greenwood M, Vali H, Titorenko VI (2013). Macromitophagy is a longevity assurance process that in
chronologically aging yeast limited in calorie supply sustains functional
mitochondria and maintains cellular lipid homeostasis.. Aging.

[71] Schroeder EA, Raimundo N, Shadel GS (2013). Epigenetic silencing mediates mitochondria stress-induced
longevity.. Cell Metab.

[72] Tahara EB, Cunha FM, Basso TO, Della Bianca BE, Gombert AK, Kowaltowski AJ (2013). Calorie restriction hysteretically primes aging Saccharomyces
cerevisiae toward more effective oxidative metabolism.. PLoS One.

[73] Martins D, Titorenko VI, English AM (2014). Cells with impaired mitochondrial H2O2 sensing generate less •OH
radicals and live longer.. Antioxid Redox Signal.

[74] Chen B, Retzlaff M, Roos T, Frydman J (2011). Cellular strategies of protein quality control.. Cold Spring Harb Perspect Biol.

[75] Lindquist SL, Kelly JW (2011). Chemical and biological approaches for adapting proteostasis to
ameliorate protein misfolding and aggregation diseases: progress and
prognosis.. Cold Spring Harb Perspect Biol.

[76] Taylor RC, Dillin A (2011). Aging as an event of proteostasis collapse.. Cold Spring Harb Perspect Biol.

[77] Kim YE, Hipp MS, Bracher A, Hayer-Hartl M, Hartl FU (2013). Molecular chaperone functions in protein folding and
proteostasis.. Annu Rev Biochem.

[78] Legakis JE, Koepke JI, Jedeszko C, Barlaskar F, Terlecky LJ, Edwards HJ, Walton PA, Terlecky SR (2002). Peroxisome senescence in human fibroblasts.. Mol Biol Cell.

[79] Terlecky SR, Koepke JI, Walton PA (2006). Peroxisomes and aging.. Biochim Biophys Acta.

[80] Ma C, Agrawal G, Subramani S (2011). Peroxisome assembly: matrix and membrane protein
biogenesis.. J Cell Biol.

[81] Liu X, Ma C, Subramani S (2012). Recent advances in peroxisomal matrix protein
import.. Curr Opin Cell Biol.

[82] Hasan S, Platta HW, Erdmann R (2013). Import of proteins into the peroxisomal matrix.. Front Physiol.

[83] Titorenko VI, Rachubinski RA (2004). The peroxisome: orchestrating important developmental decisions
from inside the cell.. J Cell Biol.

[84] Goldberg AA, Bourque SD, Kyryakov P, Boukh-Viner T, Gregg C, Beach A, Burstein MT, Machkalyan G, Richard V, Rampersad S, Titorenko VI (2009b). A novel function of lipid droplets in regulating
longevity.. Biochem Soc Trans.

[85] Ivashchenko O, Van Veldhoven PP, Brees C, Ho YS, Terlecky SR, Fransen M (2011). Intraperoxisomal redox balance in mammalian cells: oxidative
stress and interorganellar cross-talk.. Mol Biol Cell.

[86] Islinger M, Grille S, Fahimi HD, Schrader M (2012). The peroxisome: an update on mysteries.. Histochem Cell Biol.

[87] Walton PA, Pizzitelli M (2012). Effects of peroxisomal catalase inhibition on mitochondrial
function.. Front Physiol.

[88] Wang B, Van Veldhoven PP, Brees C, Rubio N, Nordgren M, Apanasets O, Kunze M, Baes M, Agostinis P, Fransen M (2013). Mitochondria are targets for peroxisome-derived oxidative stress
in cultured mammalian cells.. Free Radic Biol Med.

[89] Nordgren M, Fransen M (2014). Peroxisomal metabolism and oxidative stress.. Biochimie.

[90] Hiltunen JK, Mursula AM, Rottensteiner H, Wierenga RK, Kastaniotis AJ, Gurvitz A (2003). The biochemistry of peroxisomal beta-oxidation in the yeast
Saccharomyces cerevisiae.. FEMS Microbiol Rev.

[91] Kawałek A, Lefevre SD, Veenhuis M, van der Klei IJ (2013). Peroxisomal catalase deficiency modulates yeast lifespan
depending on growth conditions.. Aging.

[92] Lefevre SD, van Roermund CW, Wanders RJ, Veenhuis M, van der Klei IJ (2013). The significance of peroxisome function in chronological aging of
Saccharomyces cerevisiae.. Aging Cell.

[93] Minois N (2014). Molecular basis of the 'anti-aging' effect of spermidine and
other natural polyamines - a mini-review.. Gerontology.

[94] Morselli E, Galluzzi L, Kepp O, Criollo A, Maiuri MC, Tavernarakis N, Madeo F, Kroemer G (2009). Autophagy mediates pharmacological lifespan extension by
spermidine and resveratrol.. Aging.

[95] Morselli E, Mariño G, Bennetzen MV, Eisenberg T, Megalou E, Schroeder S, Cabrera S, Bénit P, Rustin P, Criollo A, Kepp O, Galluzzi L, Shen S, Malik SA, Maiuri MC, Horio Y, López-Otín C, Andersen JS, Tavernarakis N, Madeo F, Kroemer G (2011). Spermidine and resveratrol induce autophagy by distinct pathways
converging on the acetylproteome.. J Cell Biol.

[96] Binns D, Januszewski T, Chen Y, Hill J, Markin VS, Zhao Y, Gilpin C, Chapman KD, Anderson RG, Goodman JM (2006). An intimate collaboration between peroxisomes and lipid
bodies.. J Cell Biol.

[97] D'Autréaux B, Toledano MB (2007). ROS as signalling molecules: mechanisms that generate specificity
in ROS homeostasis.. Nat Rev Mol Cell Biol.

[98] Giorgio M, Trinei M, Migliaccio E, Pelicci PG (2007). Hydrogen peroxide: a metabolic by-product or a common mediator of
ageing signals?. Nat Rev Mol Cell Biol.

[99] Veal EA, Day AM, Morgan BA (2007). Hydrogen peroxide sensing and signaling.. Mol Cell.

[100] Goodman JM (2008). The gregarious lipid droplet.. J Biol Chem.

[101] Adeyo O, Horn PJ, Lee S, Binns DD, Chandrahas A, Chapman KD, Goodman JM (2011). The yeast lipin orthologue Pah1p is important for biogenesis of
lipid droplets.. J Cell Biol.

[102] Kohlwein SD, Veenhuis M, van der Klei IJ (2013). Lipid droplets and peroxisomes: key players in cellular lipid
homeostasis or a matter of fat - store 'em up or burn 'em
down.. Genetics.

[103] Sheibani S, Richard VR, Beach A, Leonov A, Feldman R, Mattie S, Khelghatybana L, Piano A, Greenwood M, Vali H, Titorenko VI (2014). Macromitophagy, neutral lipids synthesis, and peroxisomal fatty
acid oxidation protect yeast from "liponecrosis", a previously unknown form
of programmed cell death.. Cell Cycle.

[104] van der Klei IJ, Yurimoto H, Sakai Y, Veenhuis M (2006). The significance of peroxisomes in methanol metabolism in
methylotrophic yeast.. Biochim Biophys Acta.

[105] Piper PW, Harris NL, MacLean M (2006). Preadaptation to efficient respiratory maintenance is essential
both for maximal longevity and the retention of replicative potential in
chronologically ageing yeast.. Mech Ageing Dev.

[106] Lavoie H, Whiteway M (2008). Increased respiration in the sch9Δ mutant is required for
increasing chronological life span but not replicative life
span.. Eukaryot Cell.

[107] Burstein MT, Titorenko VI (2014). A mitochondrially targeted compound delays aging in yeast through
a mechanism linking mitochondrial membrane lipid metabolism to mitochondrial
redox biology.. Redox Biol.

[108] Schroeder EA, Shadel GS (2014). Crosstalk between mitochondrial stress signals regulates yeast
chronological lifespan.. Mech Ageing Dev.

[109] Broach JR (2012). Nutritional control of growth and development in
yeast.. Genetics.

[110] De Virgilio C (2012). The essence of yeast quiescence.. FEMS Microbiol Rev.

[111] Orzechowski Westholm J, Tronnersjö S, Nordberg N, Olsson I, Komorowski J, Ronne H (2012). Gis1 and Rph1 regulate glycerol and acetate metabolism in glucose
depleted yeast cells.. PLoS One.

[112] Fabrizio P, Gattazzo C, Battistella L, Wei M, Cheng C, McGrew K, Longo VD (2005). Sir2 blocks extreme life-span extension.. Cell.

[113] Burtner CR, Murakami CJ, Kennedy BK, Kaeberlein M (2009). A molecular mechanism of chronological aging in
yeast.. Cell Cycle.

[114] Burtner CR, Murakami CJ, Olsen B, Kennedy BK, Kaeberlein M (2011). A genomic analysis of chronological longevity factors in budding
yeast.. Cell Cycle.

[115] Murakami C, Delaney JR, Chou A, Carr D, Schleit J, Sutphin GL, An EH, Castanza AS, Fletcher M, Goswami S, Higgins S, Holmberg M, Hui J, Jelic M, Jeong KS, Kim JR, Klum S, Liao E, Lin MS, Lo W, Miller H, Moller R, Peng ZJ, Pollard T, Pradeep P, Pruett D, Rai D, Ros V, Schuster A, Singh M, Spector BL, Vander Wende H, Wang AM, Wasko BM, Olsen B, Kaeberlein M (2012). pH neutralization protects against reduction in replicative
lifespan following chronological aging in yeast.. Cell Cycle.

[116] Eisenberg T, Schroeder S, Andryushkova A, Pendl T, Küttner V, Bhukel A, Mariño G, Pietrocola F, Harger A, Zimmermann A, Moustafa T, Sprenger A, Jany E, Büttner S, Carmona-Gutierrez D, Ruckenstuhl C, Ring J, Reichelt W, Schimmel K, Leeb T, Moser C, Schatz S, Kamolz LP, Magnes C, Sinner F, Sedej S, Fröhlich KU, Juhasz G, Pieber TR, Dengjel J, Sigrist SJ, Kroemer G, Madeo F (2014). Nucleocytosolic depletion of the energy metabolite
acetyl-coenzyme A stimulates autophagy and prolongs
lifespan.. Cell Metab.

[117] Hu J, Wei M, Mirzaei H, Madia F, Mirisola M, Amparo C, Chagoury S, Kennedy B, Longo V (2014). Tor-Sch9 deficiency activates catabolism of the ketone body-like
acetic acid to promote trehalose accumulation and longevity.. Aging Cell.

[118] Fraenkel DG (2011). Yeast intermediary metabolism.. Cold Spring Harbor Laboratory Press, New York.

[119] Crespo JL, Powers T, Fowler B, Hall MN (2002). The TOR-controlled transcription activators GLN3, RTG1, and RTG3
are regulated in response to intracellular levels of
glutamine.. Proc Natl Acad Sci USA.

[120] Powers RW 3rd, Kaeberlein M, Caldwell SD, Kennedy BK, Fields S (2006). Extension of chronological life span in yeast by decreased TOR
pathway signaling.. Genes Dev.

[121] Jewell JL, Russell RC, Guan KL (2013). Amino acid signalling upstream of mTOR.. Nat Rev Mol Cell Biol.

[122] Conrad M, Schothorst J, Kankipati HN, Van Zeebroeck G, Rubio-Texeira M, Thevelein JM (2014). Nutrient sensing and signaling in the yeast Saccharomyces
cerevisiae.. FEMS Microbiol Rev.

[123] Shimobayashi M, Hall MN (2014). Making new contacts: the mTOR network in metabolism and
signalling crosstalk.. Nat Rev Mol Cell Biol.

[124] Swinnen E, Ghillebert R, Wilms T, Winderickx J (2014). Molecular mechanisms linking the evolutionary conserved
TORC1-Sch9 nutrient signalling branch to lifespan regulation in
Saccharomyces cerevisiae.. FEMS Yeast Res.

[125] Barea F, Bonatto D (2009). Aging defined by a chronologic-replicative protein network in
Saccharomyces cerevisiae: an interactome analysis.. Mech Ageing Dev.

[126] Lorenz DR, Cantor CR, Collins JJ (2009). A network biology approach to aging in yeast.. Proc Natl Acad Sci USA.

[127] Borklu Yucel E, Ulgen KO (2011). A network-based approach on elucidating the multi-faceted nature
of chronological aging in S. cerevisiae.. PLoS One.

[128] Gozuacik D, Bialik S, Raveh T, Mitou G, Shohat G, Sabanay H, Mizushima N, Yoshimori T, Kimchi A (2008). DAP-kinase is a mediator of endoplasmic reticulum stress-induced
caspase activation and autophagic cell death.. Cell Death Differ.

[129] Eisenberg-Lerner A, Bialik S, Simon HU, Kimchi A (2009). Life and death partners: apoptosis, autophagy and the cross-talk
between them.. Cell Death Differ.

[130] Bialik S, Zalckvar E, Ber Y, Rubinstein AD, Kimchi A (2010). Systems biology analysis of programmed cell
death.. Trends Biochem Sci.

[131] Zalckvar E, Bialik S, Kimchi A (2010). The road not taken: a systems level strategy for analyzing the
cell death network.. Autophagy.

[132] Zalckvar E, Yosef N, Reef S, Ber Y, Rubinstein AD, Mor I, Sharan R, Ruppin E, Kimchi A (2010). A systems level strategy for analyzing the cell death network:
implication in exploring the apoptosis/autophagy connection.. Cell Death Differ.

[133] Munoz AJ, Wanichthanarak K, Meza E, Petranovic D (2012). Systems biology of yeast cell death.. FEMS Yeast Res.

[134] Rubinstein AD, Kimchi A (2012). Life in the balance - a mechanistic view of the crosstalk between
autophagy and apoptosis.. J Cell Sci.

[135] Young MM, Kester M, Wang HG (2013). Sphingolipids: regulators of crosstalk between apoptosis and
autophagy.. J Lipid Res.

[136] Büttner S, Eisenberg T, Herker E, Carmona-Gutierrez D, Kroemer G, Madeo F (2006). Why yeast cells can undergo apoptosis: death in times of peace,
love, and war.. J Cell Biol.

[137] Carmona-Gutierrez D, Eisenberg T, Büttner S, Meisinger C, Kroemer G, Madeo F (2010). Apoptosis in yeast: triggers, pathways,
subroutines.. Cell Death Differ.

